# The Influence of Spatial Registration on Detection of Cerebral Asymmetries Using Voxel-Based Statistics of Fractional Anisotropy Images and TBSS

**DOI:** 10.1371/journal.pone.0036851

**Published:** 2012-06-05

**Authors:** Siawoosh Mohammadi, Simon S. Keller, Volkmar Glauche, Harald Kugel, Andreas Jansen, Chloe Hutton, Agnes Flöel, Michael Deppe

**Affiliations:** 1 Department of Neurology, University of Münster, Münster, Germany; 2 Wellcome Trust Centre for Neuroimaging, UCL Institute of Neurology, University College London, London, United Kingdom; 3 Department of Clinical Neuroscience, Institute of Psychiatry, King’s College London, London, United Kingdom; 4 Department of Neurology, Neurozentrum, University Clinic Freiburg, Freiburg, Germany; 5 Department of Clinical Radiology, University of Münster, Münster, Germany; 6 Section of BrainImaging, Department of Psychiatry and Psychotherapy, Philipps-University Marburg, Marburg, Germany; 7 Department of Neurology, Center for Stroke Research Berlin, and Cluster of Excellence NeuroCure, Charite Universitätsmedizin, Berlin, Germany; Institute of Psychology, Chinese Academy of Sciences, China

## Abstract

The sensitivity of diffusion tensor imaging (DTI) for detecting microstructural white matter alterations has motivated the application of voxel-based statistics (VBS) to fractional anisotropy (FA) images (FA-VBS). However, detected group differences may depend on the spatial registration method used. The objective of this study was to investigate the influence of spatial registration on detecting cerebral asymmetries in FA-VBS analyses with reference to data obtained using Tract-Based Spatial Statistics (TBSS). In the first part of this study we performed FA-VBS analyses using three single-contrast and one multi-contrast registration: (i) whole-brain registration based on T2 contrast, (ii) whole-brain registration based on FA contrast, (iii) individual-hemisphere registration based on FA contrast, and (iv) a combination of (i) and (iii). We then compared the FA-VBS results with those obtained from TBSS. We found that the FA-VBS results depended strongly on the employed registration approach, with the best correspondence between FA-VBS and TBSS results when approach (iv), the “multi-contrast individual-hemisphere” method was employed. In the second part of the study, we investigated the spatial distribution of residual misregistration for each registration approach and the effect on FA-VBS results. For the FA-VBS analyses using the three single-contrast registration methods, we identified FA asymmetries that were (a) located in regions prone to misregistrations, (b) not detected by TBSS, and (c) specific to the applied registration approach. These asymmetries were considered candidates for apparent FA asymmetries due to systematic misregistrations associated with the FA-VBS approach. Finally, we demonstrated that the “multi-contrast individual-hemisphere” approach showed the least residual spatial misregistrations and thus might be most appropriate for cerebral FA-VBS analyses.

## Introduction

Diffusion tensor magnetic resonance imaging (DTI) may be used to quantitatively analyse the morphology and integrity of white matter structure [1]–[7], which is of particular interest in the clinical and cognitive neurosciences [8]–[12]. Recent developments have enabled automated voxel-based statistical analyses of DTI data, so that fractional anisotropy (FA) images, for example, may be quantitatively compared between groups of subjects without manual investigator dependency, e.g. [13]–[15]. Typically either voxel-based statistics (VBS) or Tract-Based Spatial Statistics (TBSS, [15]) are used to analyse FA images in group comparison studies. An important application of such analyses is the investigation of white matter asymmetries in the healthy human brain. For example, the detection of FA asymmetries in the perisylvian region could be associated with functional language lateralization and could supplement known tractography [16]–[19] and volumetric [20] studies.

**Figure 1:**
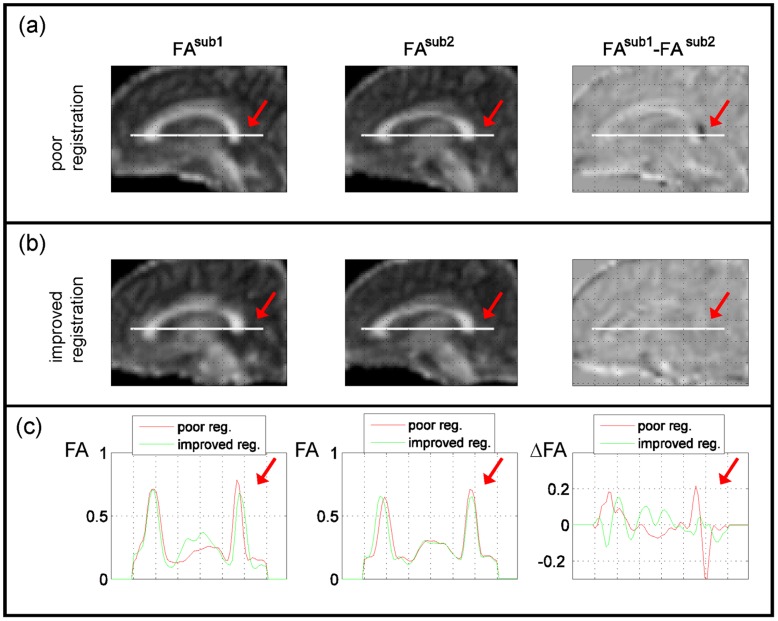
Example data from two subjects showing how FA differences are dominated by morphometrical differences when the registration is poor and how these effects are reduced when registration is improved. (a) First row shows FA images and difference image after poor registration. (b) Second row shows FA images and difference image after improved registration. (c) Third row shows profiles through FA and ΔFA maps (along white line in (a) and (b)). Arrow highlights region that is dominated by FA difference due to misregistration when the registration is poor (a).

VBS of FA images (FA-VBS) preserves the complete white matter architecture of the brain and allows for the comparison of FA values in corresponding regions across subjects. However, depending on the registration approach the inter-individual FA differences in certain voxels, particularly towards the edge of white matter pathways, can originate predominately from differences in the *local morphometry* between subjects (see Figure 1) rather than from microstructural differences. A major objective of the present study was to investigate the influence of different spatial registration approaches on residual morphometric differences between registered FA maps.

Various methods have been proposed for spatial registration of DTI data (see e.g. Table 1). One often used registration approach is based on structural T1-weighted (T1w) images (e.g. [2]), for which established and optimized software packages are available (e.g., DARTEL ([21]). However, since DTI images are usually acquired using echo planar imaging (EPI), they are distorted due to susceptibility artefacts [22] and therefore do not align with T1w images. In this case additional measurements are necessary to estimate and correct for the EPI distortions [23], [24]. Alternatively, image contrasts based only on DTI images can be used such as, T2-weighted (T2w) images measured without diffusion gradient (i.e. with b = 0 s/m2, here denoted as the b0 image) or FA images, which require no additional data to correct for EPI distortions. Furthermore, it has been hypothesized that registration of DTI data could be improved if multiple contrasts, e.g. b0 and FA, were used for spatial registration [25]-[28]. Finally, it was previously reported that in an analysis of cerebral asymmetry fewer misregistrations occurred when the individual hemispheres were separated and independently spatially normalised [13].

**Table 1:** Studies that differ by the registration contrast employed for voxel-based statistics of DTI data.

Registration contrast (template)	DTI	Subjects/Patients	Topic	Reference
T1 (SPM)	1.5T GE, EPI, 20 directions	20 schizophrenia, 24 controls	FA, ADC, grey matter differences	Agartz et al. [67]
T1 (SPM)	1.5T S, STEAM, 6 directions	15 stuttering, 15 controls	FA differences	Sommer et al. [68]
T1 (SPM)	1.5T GE, EPI, 41 directions	11 fast learners vs. 10 slow learners	FA and grey matter differences	Golestani et al. [69]
b0 (customized)	1.5T S, EPI, 6 directions	14 PSP, 14 controls	FA differences	Padovani et al. [70]
b0 (customized)	1.5T GE, EPI, 64 directions	14 schizophrenia, 14 controls	FA differences/smoothing kernel	Jones et al. [71]
b0 (SPM)	1.5T GE, EPI, 25 directions	18 Cadasil	Correlation: FA vs. executive functions	O’Sullivan et al. [72]
b0* (customized)	1.5T S, 24 directions	54 healthy	Correlation: ADC and RA vs. age	Camara et al. [73]
FA* (customized)	1.5T S, STEAM, 6 directions	9 left handed healthy, 19 left handed healthy	FA differences	Buchel et al. [13]
FA (customized)	1.5T S, EPI, 12 directions	84 healthy volunteers	Correlation: FA vs. age	Pagani et al. [74]
FA (customized)	1.5T S, EPI, 6 directions	24 schizophrenia, 24 controls	FA differences	Caan et al. [75]
Multi-contrast (b0, **D**)	1.5T GE, LSDI, 6 directions	23 schizophrenia, 32 controls	FA differences	Park et al. [14]
Multi-contrast (b0, FA)	3T P, EPI, 20 directions	18 Epilepsy patients, 67 controls	FA differences,Correlation: FA vs. seizure frequency	Deppe et al. [10]

Abbreviations: SPM  =  stereotactic space provided by SPM, D  =  diffusion tensor, P  =  Philips, S  =  Siemens, GE  =  General Electric, STEAM  =  Stimulated Echo Acquisition Mode, EPI  =  Echo Planar Imaging, LSDI  =  Line Scan Diffusion Imaging, PSP  =  Progressive Supranuclear Palsy, ADC  =  Apparent Diffusion Coefficient, RA  =  Relative Anisotropy, (*)  =  optimized normalization process.

To validate detected group left-right FA differences in VBS analyses and disentangle the morphometric and microstructural differences a gold standard such as postmortem neuroanatomical assessment would be desirable (see e.g. [29]). In the absence of such a gold standard, we compared the results of FA-VBS analyses with TBSS [15], which uses an FA skeleton for normalisation and spatially restricting the statistical analysis to the tract centre. As a result of the restriction to the tract centre, TBSS is less prone to FA differences due to misregistrations which predominantly occur at the edge of white matter pathways (Fig. 1). Although residual morphometric differences are eliminated with TBSS, it should not be considered as a gold standard. In particular, the sensitivity of TBSS for detecting FA differences beyond the central white matter pathways (e.g. in deep gray matter nuclei [30]) might be reduced relative to the FA-VBS method.

The present study is divided in two parts. In Experiment I, we investigated the role of registration in FA-VBS analyses and compared the statistical maps to TBSS results. We performed FA-VBS analyses to identify hemispheric asymmetries using four different registration procedures: (i) whole-brain registration based on the b0 contrast, (ii) whole-brain registration based on the FA contrast, (iii) individual-hemisphere registration based on the FA contrast, and (iv) a combination of (i) and (iii). In Experiment II, we evaluated the spatial location and amount of misregistrations of the image registration approaches (i) to (iv). To assess misregistration, we labelled the relevant white matter (WM) using FA masks that were free of microstructural information and thus solely reflected morphological differences. Finally, we compared the statistical maps revealed by FA-VBS to regions prone to misregistration using TBSS maps as an additional reference in order to identify apparent FA asymmetries due to systematic misregistrations.

## Methods

### Ethics

This study was carried out in strict accordance with the principles expressed in the Declaration of Helsinki. The study was approved by the ethics committee of the Medical Association Westphalia/Lippe and the Medical Faculty of the University of Muenster. All participants provided written informed consent before the scanning sessions.

### Subjects

Nineteen neurologically and psychiatrically healthy right-handed volunteers (8 females; median age 28 years, range 20–39 years) were included in this study. Standard exclusion criteria for MR imaging were applied.

### Data Acquisition and Estimation of the Diffusion Tensor

Data were acquired using a Gyroscan Intera 3T whole body MRI system (Philips, Best, the Netherlands) with a transmit/receive birdcage head coil and maximum gradient amplitude of 33 mT/m. The DTI data were acquired in 36 axial slices 3.6 mm thick with no gap, quadratic field of view 230 mm x 230 mm, acquired matrix 128 x 128, reconstructed to 256 x 256 after zero filling, resulting in a voxel size of 1.8 mm x 1.8 mm x 3.6 mm measured, and 0.9 mm x 0.9 mm x 3.6 mm after reconstruction (right-left (x); anterior-posterior (y); inferior-superior directions (z)). The echo time was 95 ms and the repetition time was 9473 ms. A b-value of 1000 s/mm^2^ was used for a total of 20 diffusion-weighted (DW) images, with isotropic gradient directions [31]. Each DW image was measured twice and averaged to one single DW image. The acquisition of the non-diffusion weighted image (b0 image) was repeated six times, all the b0 images were also averaged to a single image. In sum, 21 images per slice were used for diffusion-tensor calculation. The total data acquisition time was approximately 8 minutes per subject. First, the DTI data were visually checked for obvious imaging artefacts using the residual error map of the tensor fit to detect outliers [32], [33]. After ensuring that no visible imaging artefacts (e.g. vibration artefacts [34], [35]) were detected by the residual error of the tensor fit, the DTI data were corrected for motion and eddy current effects using an in-house software written in MATLAB (version 7.11.0; Mathworks, Natick, MA, USA) [36]. Finally, FA values were generated from the pre-processed DTI data using FMRIB’s Diffusion Toolbox [37].

### Spatial Registration

Four different registration approaches were used: three were based on single-contrasts (either b0 or FA) and one on multi-contrast (FA and b0). The default settings of the SPM8 normalisation software were used for each registration approach [38], except for the smoothing of the template; the latter was set to the smoothing kernel of the source image (FWHM  = 8mm). A set of 7 x 9 x 7 basis functions in *x*-, *y*-, and *z*-direction was used for the parameterization of the non-linear transformations. Common to all four registration methods was that the b0 image had been first coregistered using an *affine* transformation to the standard SPM EPI template. The same transformation was subsequently applied to the corresponding FA image and to the DW images. Table 2 summarises the three single-contrast registration approaches that used different source images, selection masks (i.e. masks using to identify whole-brain or single hemispheres), and customized templates. In order to minimize the effect of the non-smooth transition from 'brain' to 'non-brain' at the edges of the selection masks the source images were smoothed after masking.

**Table 2:** The three single-contrast registrations using different templates, source-images and selection masks.

Registration approach	(i)	(ii)	(iii)
Contrast	b0	FA	FA
Selection mask	none	whole-brain	separate hemisphere
Source	whole brain b0 image	whole brain FA image	left and flipped right hemisphere of FA image
Template	customised average of whole brain b0 images	customised average of whole brain FA images	customised average of individual hemispheres of FA images

For details see text.

#### Registration approach (i)

Source and template were constructed from b0 images. The subjects’ individual b0 images (T2 contrast) were used as source images. No selection mask was used for this registration approach.

#### Registration approach (ii)

Source and template were constructed from FA images. The whole brain selection mask was used to set FA values to zero in voxels not belonging to the brain tissue.

#### Registration approach (iii)

Source and template were constructed from FA images. Hemispheres were registered separately using the hemisphere selection mask. FA values in the unconsidered hemisphere were set to zero. In a second step the previously unconsidered hemisphere was registered by flipping the original FA image and repeating the registration process.

#### Registration approach (iv)

The multi-contrast approach consisted of the successive application of approaches (i) and (iii). In the first step the whole brain was registered using approach (i) based on the T2 image contrast. In the second step individual hemispheres were registered using the separate-hemisphere registration (approach (iii)).

For each registration approach we used a left-right symmetrized, customized template calculated from the normalised FA or b0 images. To minimize the influence of image blurring (ill-defined anatomic contrast in the customized FA and b0 templates) on the registration accuracy, the templates were calculated iteratively. First, we calculated a preliminary template using the affine transformed (b0 or FA) images. Second, images were registered to this preliminary template using nonlinear normalisation. Thirdly, we recalculated a further preliminary template using the normalised images until the influence of the template on the registration procedure was negligible (i.e. the difference between the generated templates were below 2%). The template generation procedure and the registration approaches (i-iv) are part of our FA-VBS normalisation toolbox [39], [40] that can be found within the diffusion toolbox II of SPM8 (http://spmtools.svn.sourceforge.net/viewvc/spmtools/tbxDiffusion/) and a newer version can be downloaded as a separate toolbox (http://www.fil.ion.ucl.ac.uk/spm/ext/).

### Experiment I: Influence of Spatial Registration on FA-VBS Results using TBSS as Reference

#### FA-VBS and TBSS analyses

In the first analysis we investigated the influence of four registration approaches (i - iv) on detecting cerebral asymmetries via FA-VBS. In addition we performed a TBSS analysis of hemispheric asymmetry and used TBSS results as a reference (see Table 3). It was sufficient to analyse left-greater-than-right effects only, because no substantially new information about misregistrations could be gained by the analysis of the opposite effect (right-greater-than-left).

**Table 3:** Processing steps for (a) FA-VBS [39], [40] and (b) TBSS (http://fsl.fmrib.ox.ac.uk/fsl/tbss/index.html).

Steps	(a) FA-VBS	(b) TBSS
Pre-processing I	prepare data (step zero)	prepare data (tbss_1_preproc)
Non-linear registration	single- or multi-contrast registration approach (i-iv)	single-contrast FA-based (tbss_2_reg)
Smoothing	FWHM = 4mm (isotropic)	none
Pre-processing II	mask smoothed images byFA mask (threshold: FA>0.2)	create mean FA image and skeletonisation (tbss_3_postreg); project FA data onto mean FA skeleton (tbss_4_prestats)
Statistics	paired t-test of original and flipped FA images	paired t-test of original and flipped FA images

1. The FA-VBS protocol consists of three processing steps:
  1. Registration: The original and flipped FA maps were registered to symmetrized, customized templates using registration approaches (i) to (iv) (see also section *Spatial registration*).
  2. Smoothing: The normalised FA maps were smoothed with a kernel size of 4 mm FWHM.
  3. Inference statistics: The FA-VBS was performed by means of a paired *t*-test. We tested the null hypothesis H_0_ =  “no inter-hemispheric FA differences” on a voxel-by-voxel basis (significance level: p<0.01 and p<0.001, uncorrected). To exclude low FA values, which are particularly susceptible to noise [41], [42], we multiplied the subjects’ FA maps by an FA mask which was generated from the symmetrized, group averaged, normalised FA maps (thresholded at FA values >0.2). The threshold is motivated from tractography studies, e.g., see [37].
2. The TBSS protocol was applied to the original and the flipped FA images according to the pre-processing steps of TBSS (http://fsl.fmrib.ox.ac.uk/fsl/tbss/index.html, also see Table 3). We used the left-right symmetrized, customized FA template that was generated by the FA-based registration approach (ii). The processed original and flipped FA images were compared using the FSL randomise toolbox (paired *t*-test, p<0.05, corrected for multiple comparisons across space using “threshold-free cluster enhancement” as recommended on the FSL webpage [43], 5000 permutations).

#### Quantitative comparison of FA-VBS and TBSS statistical maps

**Figure 2:**
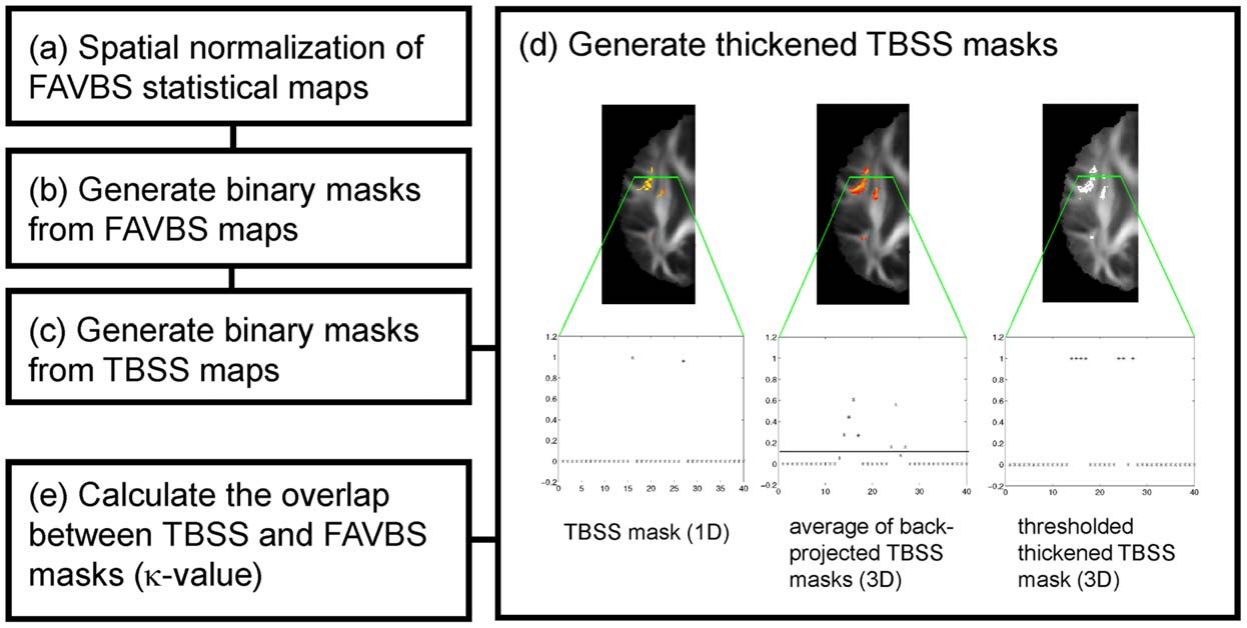
Overview of the five-step procedure to assess the overlap between FA-VBS and TBSS maps. For details see section *Quantitative comparison of FA-VBS and TBSS statistical maps*.

To compare FA-VBS and TBSS results, we determined the spatial overlap between the TBSS and FA-VBS statistical maps using a five-step procedure (Fig. 2):

In step (a), we normalised the FA-VBS-derived statistical maps to TBSS standard space. To estimate the deformation parameters, we normalised the customized FA templates of each registration approach (i-iv) to the customized FA template in the TBSS standard space. Note that although the same FA template was used in the TBSS and FA-VBS analysis, the resulting registered images were in different normalisation spaces. During the TBSS analysis the FA template was by default normalised to MNI space, whereas in the FA-VBS analysis it remained in the customized space. To correct for this mismatch the final normalisation step was necessary.

In step (b), we generated a binary FA-VBS mask for each registration method that identified regions with left-greater-than-right differences by thresholding the FA-VBS-derived statistical maps. To test whether the results depend on the p-value we used a liberal (*T*_FAVBS_ >2.6, i.e. p<0.01) and a conservative (*T*_FAVBS_ >3.7, i.e. p<0.001) threshold.

In step (c), we generated a mask that covered regions with left-greater-than-right differences by thresholding the TBSS-derived skeleton-restricted statistical map (using the recommended parameters, see section *FA-VBS and TBSS analyses*).

In step (d), we thickened the quasi-one-dimensional TBSS mask in order to increase the overlapping area of voxels reported as significant by TBSS and FA-VBS. To thicken the TBSS mask, we used the first step of the TBSS “back projection” option (http://www.fmrib.ox.ac.uk/fsl/tbss/index.html). With this option each skeleton voxel was projected back from its position on the skeleton to the nearby position at the centre of the nearest tract in the subject's FA image in standard space (i.e., after the FA image had been nonlinearly registered to the target image, see Fig. 2d, left). After back-projection, the group average of the resulting masks was calculated revealing a three-dimensional probability map (Fig. 2d, middle). Within this probability map, a voxel value of one indicates that at this location all subjects show significant left-greater-than-right FA differences, whereas a voxel value of zero states that at this location no subject shows left-right differences. To calculate the thickened TBSS mask we thresholded the probability map using different thresholds. Since the results were independent for thresholds between 0.2 and 0, we used the value of 0.1 (Fig. 2d, right) in this study.

In step (e), we calculated the weighted overlap (κ-value) between the thickened TBSS mask 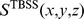, and the FA-VBS mask 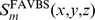:


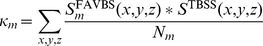
, where *x, y, z* are the coordinates of all voxels in the brain, *m* indicates the registration method (i-iv) and *N_m_* is the number of voxels for which 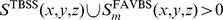. The resulting 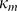 value indicates the similarity between the TBSS results and FA-VBS results for the method *m*. When 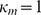 there is total overlap between TBSS and FA-VBS results, when 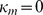 there is no overlap.

### Experiment II: Assessment of Misregistrations

To assess misregistrations we used FA masks, which were free of microstructural information and thus solely reflected morphological differences. In other words, we assumed a registration to be perfect if the masks of two given FA images showed perfect overlap after registration. We used two different methods to assess the misregistrations. The first method (section *Pairwise comparison of misregistrations by the variance maps*) was a voxelwise comparison of FA masks from pairs of registration approaches yielding a spatial map of regions susceptible to misregistrations. The second method (2.5.2, Hemispheric misregistrations) was an assessment of the spatially averaged amount of misregistrations between hemispheres of individual subjects for each single registration approach.

#### Pairwise comparison of misregistrations by the variance maps

To compare misregistrations, we first created an FA mask 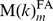 for each subject *k* and each registration method *m*  =  (i-iv) by thresholding the corresponding FA maps (FA >0.2). We then calculated the voxelwise variance of the registered FA masks over all subjects:



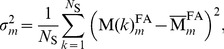
(2) where 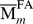 is the group average of all 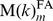, and *N*_S_ is the number of subjects.

Then we defined the symmetrized variance (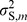) using a voxelwise sum of the variances for the left and right hemisphere: 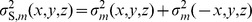


Finally, we calculated for each pair of registration methods the quotients of variance maps (i.e. a total of 12 quotients, see Table 4). These quotients of variance maps correspond to maps of *F*-ratios 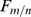 (as used in Fisher’s F Test [44]), for all combinations of registration approaches *m* and *n*. For voxels with an F-ratio 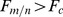, the variance of registration approach *m* was significantly greater than that of approach *n. F*_c_ is the critical *F* value as defined in statistical textbooks, e.g., [44]: *F*_c_  = 2.27, *F* distribution for 17 degrees of freedom (p<0.05). The resulting map of thresholded *F*-ratios 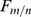 could be considered as a map identifying regions prone to misregistrations using approach *m* with approach *n* as a reference.

**Table 4:** *F*-parameters.

registration approaches	(i)	(ii)	(iii)	(iv)
(i)		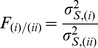	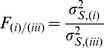	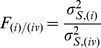
(ii)	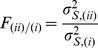		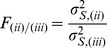	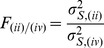
(iii)	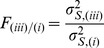	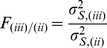		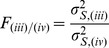
(iv)	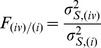	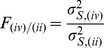	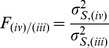	

In analogy to the *F* parameter used in Fisher’s *F* Test [44], we considered the variance in the nominator as significantly greater than the variance in the denominator when the *F*-ratio exceeded a critical *F* value (*F*_c_) (here 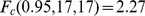, see *F* distribution for 17 degrees of freedom, *p*<0.05).

Finally, for each pair of registration approaches, we summed over all *F*-values where 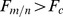 and divided by the total number of non-zero voxels in the *F*-ratio map:



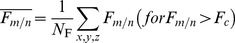
(3)


#### Hemispheric misregistrations

**Figure 3:**
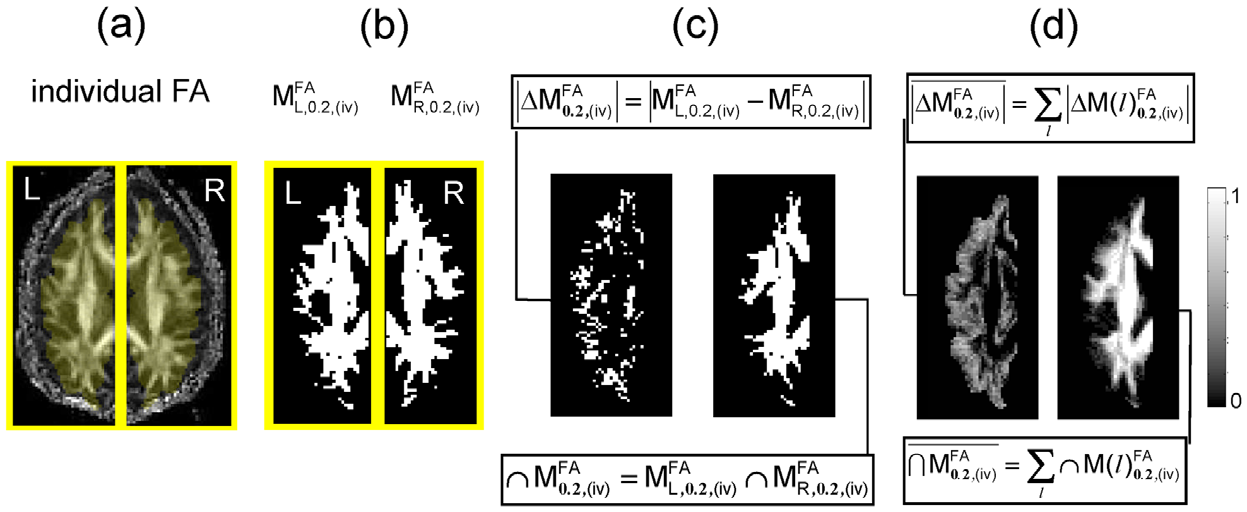
The procedure for calculating the 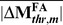 and 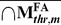 maps showed for the registration approach (iv) and the FA threshold: FA >0.2. (a) The FA image of an individual subject was thresholded (FA >0.2) and masked with an external FA mask that was also used for the FA-VBS statistics (section *FA-VBS and TBSS analysis*). (b) The left and right hemisphere of the thresholded and masked FA image for the individual subject (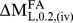 and 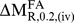). (c) The resulting difference (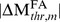) and intersection (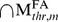) maps for the individual subject. (d) The subject-averaged 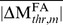 and 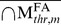 maps.

To quantify the extent of hemispheric misregistrations, we used left-right differences of FA masks (Fig. 3a and b). We first calculated the absolute value of left-right differences of individual FA masks (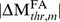) after employing registration approaches m  =  (i) - (iv) (Fig. 3c). Secondly, we calculated the intersection of the left and right hemisphere of individual FA masks (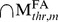, Fig. 3c). Finally, we calculated the group-average of the 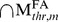 maps (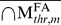, Fig. 3d) and the group average of the individual 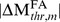 maps (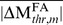, Fig. 3d). Using the 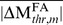 and 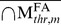 maps, we calculated a κ*_thr_* value over the entire brain for different FA mask thresholds (*thr*  = 0.2, 0.3, 0.4 and 0.5) and after employing different registration approaches (*m*  =  (i-iv)):


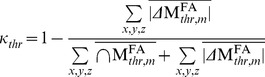
, where *x,y,z* are the coordinates of all voxels in the brain. We varied the thresholds of the FA masks to investigate the dependence of our results on the choice of threshold. The κ*_thr_* value is between zero and one, where κ*_thr_*  = 1 refers to no misregistration, i.e. total overlap between hemispheres, and κ*_thr_*  = 0 refers to maximal misregistration, i.e. no overlap.

#### Comparing group statistical results to regions susceptible to misregistrations

We compared the group statistical results, i.e. the statistical maps from FA-VBS and TBSS, to regions that were susceptible to misregistrations as identified by the maps of *F*-values (see previous section). For this purpose, we first thresholded the *F*-maps that compare the single-contrast registrations *m*  =  (i), (ii), (iii) to the multi-contrast registration (iv):



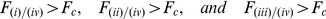
(5)


We used the multi-contrast registration (iv) as reference, because it produces the smallest amount of misregistration (see results section). Next, we projected the thresholded *F*-maps onto the FA template. Furthermore, we projected the FA-VBS statistical maps using approach *m* onto the FA template, to relate them to the misregistrations of approach *m* (given by the *F_m_*_/(*iv)*_-map). Then, we projected the TBSS statistical map (as an independent reference map) onto the FA template. FA asymmetries, which were detected only by the FA-VBS statistical map (but not by the TBSS statistical map) and were located in regions susceptible to misregistration, are possible candidates for FA-VBS results related to registration errors. Finally, we quantified the number of voxels that were detected by the FA-VBS statistical maps but not by the TBSS statistical maps for the single-contrast registrations (*m*  =  (i), (ii), (iii)):



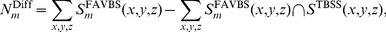
(6) and compared them to the number of voxels susceptible to misregistrations 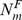 (see Eq. (5)).

## Results

### Experiment I: Influence of Spatial Registration on FA-VBS Results using TBSS as a Reference

#### The FA-VBS and TBSS-derived statistical maps

**Figure 4:**
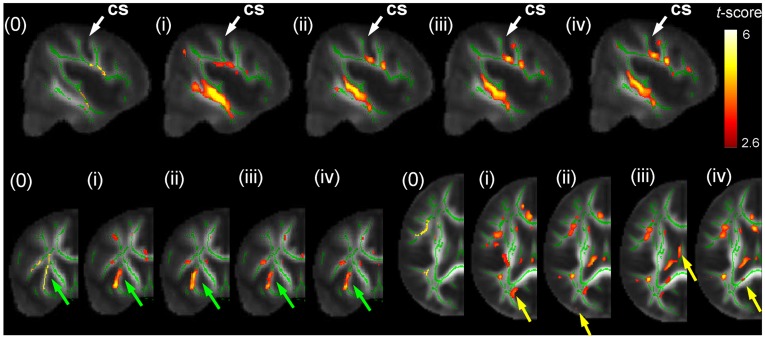
Left-greater-than-right FA differences detected by TBSS statistical maps (0) (significance level: p<0.05, corrected), and by FA-VBS statistical maps employing registration approach (i) to (iv) (significance level: p<0.01, uncorrected). For anatomical orientation the statistical maps (*t*-scores in jet colours) were projected onto the FA template (grey) overlaid by the TBSS skeleton (green). Green arrows illustrate voxels that correspond between TBSS and FA-VBS maps, yellow arrows illustrate voxels that were not detected by the TBSS approach and thus might be candidates for FA asymmetries related to systematic misregistrations (see also Fig. 8). Top line: sagittal slices (*x* = -45mm, CS  =  central sulcus); bottom line: coronal (*y* = 9mm) and planar (*z* = 19mm) slices.

Left-greater-than-right hemispheric group FA asymmetry is presented in Figure 4 using TBSS and FA-VBS statistical maps. Consistent FA asymmetries were observed between TBSS and FA-VBS results (e.g. see green arrows in Figure 4.), although some clear differences were also visible (e.g. see white and yellow arrows in Figure 4). Clear differences were also observed between the different FA-VBS statistical maps obtained using registration approaches (i) to (iv) (e.g. see yellow arrows in Figure 4). Assessed by visual inspection, the greatest correspondence between TBSS and FA-VBS maps was achieved when the separate-hemisphere multi-contrast approach (iv) was used.

#### Quantitative comparison of FA-VBS- and TBSS-derived statistical maps

**Figure 5:**
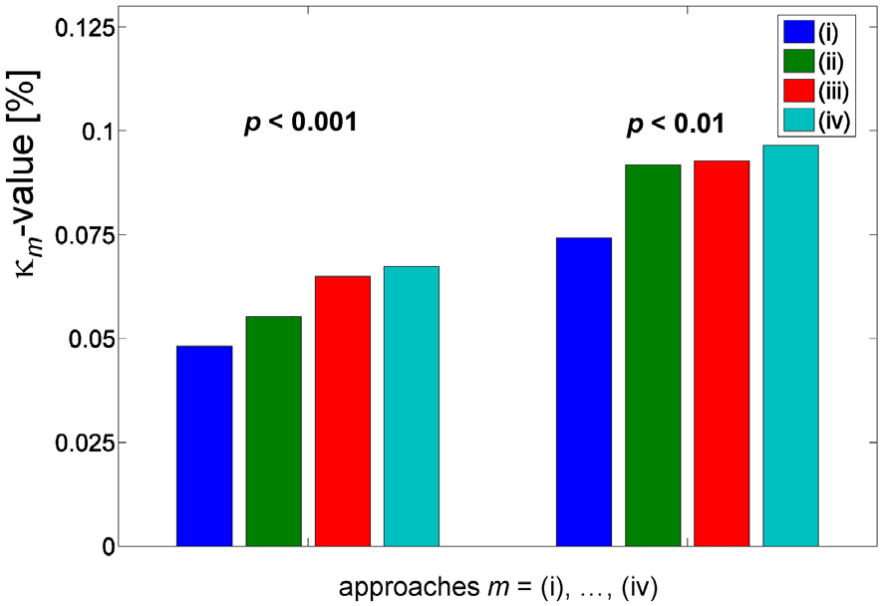
The spatial overlap between FA-VBS and “thickened” TBSS mask over the entire brain. The spatial overlap is quantified by the κ-value (see Eq. [1] and Fig. 2). The overlap was calculated for two different sets of FA-VBS masks, the first set was generated at p<0.01 and the second at p<0.001.

A plot of the κ*_m_* value representing the spatial overlap between FA-VBS and TBSS results is shown in figure 5 (*m*  =  (i),…,(iv)). The greatest overlap (κ*_m_* value) was achieved when the separate-hemisphere multi-contrast registration approach (*m* = (iv)) was used. This result was evident for different statistical significance levels employed for FA-VBS (p<0.01 and p<0.001).

### Experiment II: Assessment of Misregistration

#### Evaluating systematic misregistrations by pairwise comparison of the variance maps

**Figure 6:**
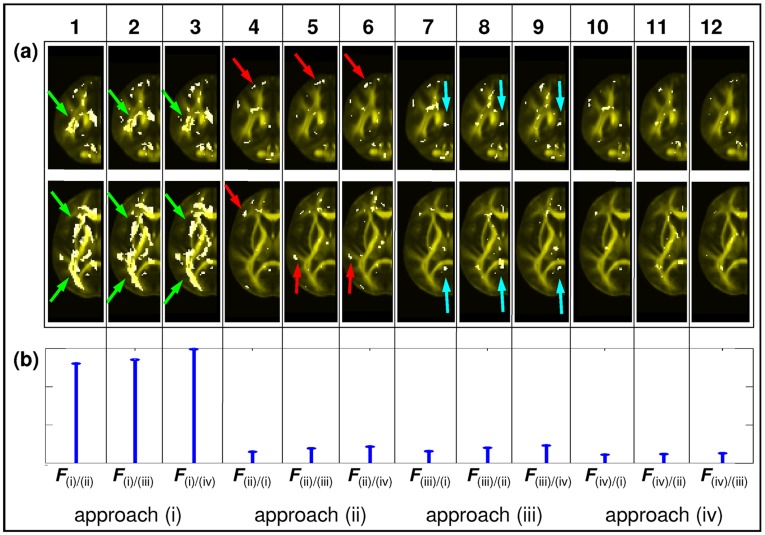
Misregistration is assessed using *F* maps (ratio of a pair of variance maps, see Table 4). (a) The thresholded *F* map **(** *F* >*F*_c_ = 2.27) is shown in white projected on the FA template (in yellow). White regions show where the registration approach in the numerator of the *F* map produces significantly more misregistrations than the approach in the denominator. Axial and coronal slices through example regions are shown. Each column shows the comparison of one approach with each of the other three; columns 1-3 show (i) compared with (ii)-(iv), columns 4-6 show (ii) compared with (i),(iii)-(iv), columns 7-9 show (iii) compared with (i)-(ii),(iv), and columns 10-12 show (iv) compared with (i)-(iii). (b) Each column shows the corresponding normalised sum of the thresholded *F* values calculated over the entire brain (see Eq. [3]).

The pairwise comparisons of the variance maps of normalised FA masks are shown in Fig. 6a. The numbers in parentheses indicate the respective registration approaches that were compared. The comparison of approach (i) to approaches (ii-iv) are presented in columns 1-3, and indicate that the variance was significantly greater in WM regions associated with the central fiber bundles if approach (i) was used (e.g. see green arrow). The comparison of approach (ii) to approaches (i), (iii) and (iv) are presented in columns 4-6 and indicate that no region had greater variance adjacent to the posterior central fiber bundles but the variance was greater adjacent to the anterior part of the corpus callosum and within lateral WM structures if approach (ii) was used (e.g. see red arrow). Comparison between approach (iii) and approaches (i), (ii) and (iv) are presented in columns 7-9. No regions with greater variance were found in lateral WM structures, but the variance was significantly greater next to the midsagittal plane if approach (iii) was used (e.g. see blue arrow). Finally, the comparison of approach (iv) to approaches (i), (ii) and (iii) are presented in columns 10-12. When compared to the whole-brain registrations the results were similar to the results presented in column 8, indicating that the variance was significantly greater next to the midsagittal plane. However, when compared with the individual-hemisphere registration fewer regions with greater variance were observed. Overall, the multi-contrast separate-hemisphere approach (iv) showed the least misregistrations, i. e. lowest normalised sum of F-values (Fig. 6b).

#### Hemispheric misregistrations

**Figure 7:**
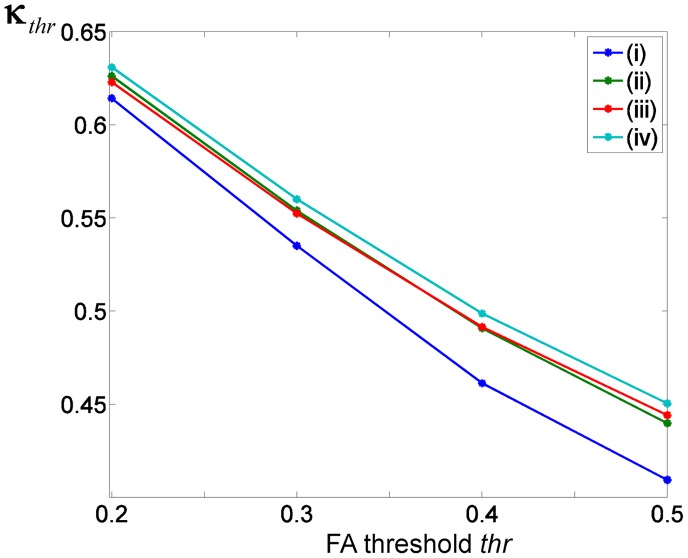
The amount of hemispheric misregistrations spatially averaged over the entire brain using the κ*_thr_* value (see Eq. [**4**]) for the different registration approaches coloured in (i) dark blue, (ii) green, (iii) in red, (iv) light blue. The κ*_thr_* value is depicted for different FA thresholds (thr  = 0.2,…,0.5) to show the FA-threshold-dependence of the κ*_thr_* value. The greater the κ*_thr_* value the smaller the amount of misregistrations between left and right hemisphere.

The misregistrations between left and right hemispheres of the normalised FA images are shown in Figure 7. They were lowest (i.e. largest κ*_thr_* values) when the separate-hemisphere multi-contrast registration approach (iv) was used (light blue line) and smallest when the registration approach (i) was used (dark blue line, Figure 7). The κ*_thr_* values of the registration approaches (ii) and (iii) were most similar (red and green line). Overall, approach (iv) was consistently better than other approaches at all FA thresholds and approach (i) was consistently worse than all other approaches.

#### Comparing group statistical results to regions susceptible to misregistrations

**Figure 8:**
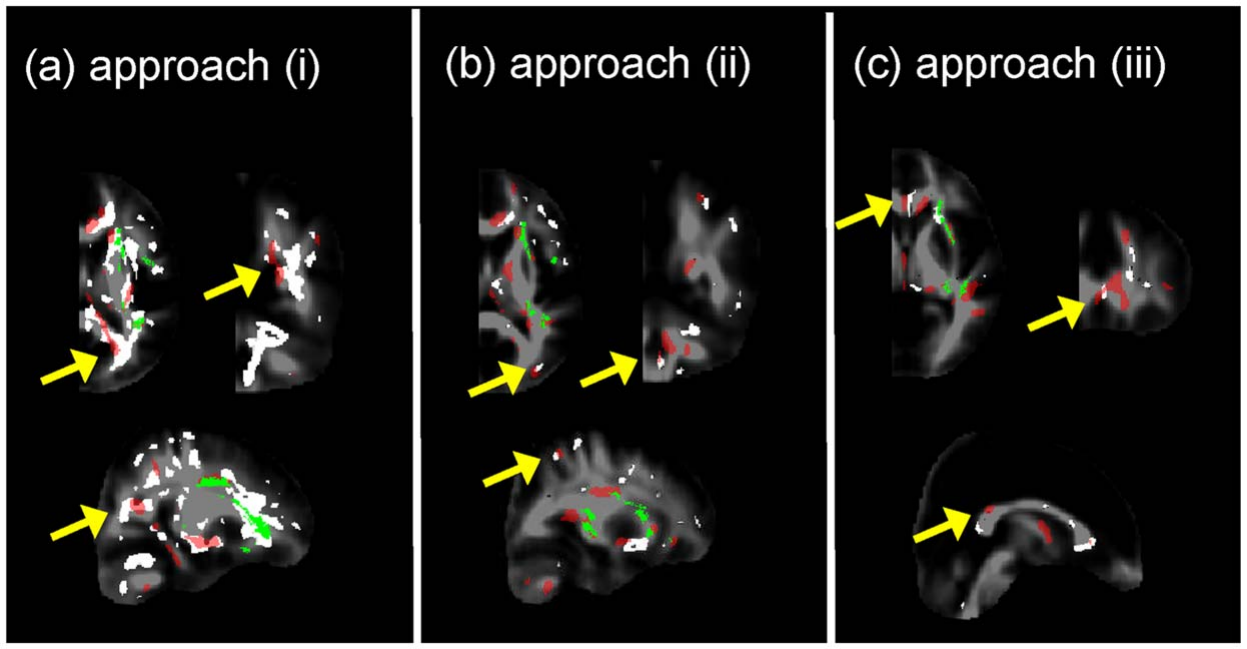
Examples of FA asymmetries (arrows) revealed by FA-VBS (red maps) that were specific to the applied registration approach, were not detected using TBSS (green maps), and located in regions prone to misregistrations (white maps). FA-VBS maps were calculated for each of the single-contrast registration approaches (approach (i), Fig. 8a, approach (ii), Fig. 8b, and approach (iii), Fig. 8c) and regions prone to misregistrations were identified by thresholded F-maps (white) comparing the corresponding single-contrast registration with the more accurate multi-contrast, separate-hemisphere registration (iv): *F*_(i)/(iv)_, *F*_(ii)/(iv)_, and *F*_(iii)/(iv)_. For anatomical orientation all maps were projected onto the FA template (grey scale).

Figure 8 shows examples of FA asymmetries (arrows) revealed by FA-VBS that were (a) specific to the applied registration approach, (b) not detected using TBSS, and (c) located in regions prone to misregistrations. A candidate for apparent FA asymmetries detected by FA-VBS using registration approach (i) was located at the edges of WM structures associated with the fiber bundles at the posterior part of the corpus callosum (arrows, Fig. 8a). FA-VBS employing approach (ii) revealed a candidate for apparent FA asymmetries located along the borders of lateral WM structures that overlapped with a region susceptible to misregistrations (arrows, Fig. 8b). When the hemispheres were registered separately by approach (iii) a candidate for apparent FA asymmetry was detected along the midsagittal plane that overlapped with a region susceptible to misregistrations (arrows, Fig. 8c).

**Figure 9:**
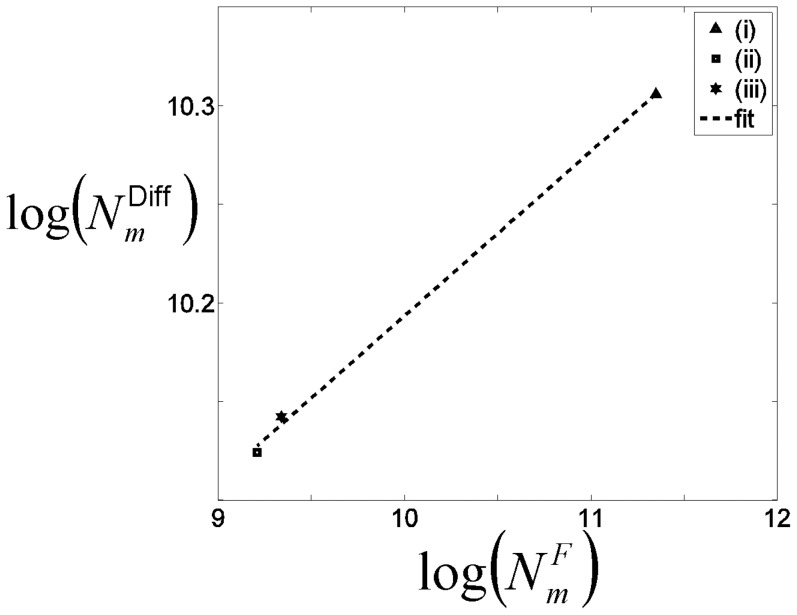
Figure 9 compares for each single-contrast registration approach *m*  =  (i), (ii), (iii), the number of voxels that were detected via the FA-VBS but not via TBSS statistical maps (*N_m_* ^Diff^) to the number of voxels that were susceptible to misregistrations (*N_m_^F^*). The number of voxels susceptible to misregistration was determined by the thresholded F-maps that correspond to the *m-*th registration approach. The number of voxels that show a mismatch between TBSS and FA-VBS were calculated by Eq. (6). To emphasis the relation between *N_m_* ^Diff^ and *N_m_^F^*, the data is depicted in log-scale and a straight line is fitted to the data.

Figure 9 compares for each single-contrast registration approach the number of voxels that are detected via the FA-VBS but not via TBSS statistical maps to the number of voxels that are susceptible to misregistrations. The number of voxels that are susceptible to misregistrations and different between the FA-VBS and TBSS results were greater for the b0-based approach (i) compared to the FA-based approaches (ii) and (iii) (Fig. 9). Averaged over the whole brain, the number of voxels that are susceptible to misregistrations increased with the number of voxels that are different between the FA-VBS and TBSS results (Fig. 9).

## Discussion

In the present study, we compared the influence of four different image registration approaches on the detection of WM asymmetries in healthy right-handed subjects using FA-VBS with reference to TBSS. Novel FA asymmetries emerged for each registration approach. The greatest similarity between TBSS and FA-VBS in detecting left-greater-than-right differences was observed when the multi-contrast separate-hemisphere registration approach (iv) was employed. This approach also resulted in the least between-subject and between-hemisphere misregistrations.

### Disentangling FA Differences Due to Misregistration from Microstructural Differences

FA-VBS statistical maps reflect residual morphological as well as microstructural multi-subject differences. One approach to disentangle these differences is to measure residual misregistrations using binary masks, which are free of microstructural information and thus only reflect morphological differences. Previous studies assessing misregistrations used anatomical structures in T1w images to label morphology and calculate a measure of overlap of the normalised labelling masks [45]-[48]. In EPI-based DTI, however, the individually varying misalignments between T1w and DTI images due to susceptibility artefacts would require additional image acquisition and preprocessing [23], [24]. In this study, we used binary masks based on FA maps. Compared to masks derived from tractography [48] our approach avoids using an additional method with potentially new confounds (e.g. the tractography algorithm and associated issues with anisotropic image resolution as well as determination of seed points in individual subjects and hemispheres). The FA threshold to create binary masks must be motivated in a similar way to tractography-based methods [37], [48]. For the pairwise comparison of registration approaches (Fig. 6) we used the same FA threshold as for masking the FA-VBS results (*thr*  = 0.2, a typical threshold used in tractography studies [37]). For the assessment of the hemispheric misregistrations we explored a range of FA thresholds and showed that our main results (misregistrations were highest in approach (i) and lowest in approach (iv)) were independent of the FA threshold selection (Fig. 7).

### Assessment of Misregistrations

Previously it has been shown that the registration accuracy is strongly dependent on the image contrast [26]. For each of the whole-brain single-contrast registration approaches ((i) and (ii)) we showed that the majority of voxels prone to misregistrations were located in brain areas where the tissue contrast was poor in the image used to drive the registration (e.g. central WM for b0 and cortex for FA, column 1 and 4 in Fig. 6a). Our results suggest that the separate registration of individual hemispheres (approaches (iii) and (iv)) improves registration accuracy particularly for regions with opposite deformations (e.g. increasing the size of the left ventricle while independently decreasing the size of the right ventricle). We attribute this to the inherent global anatomical shape differences between the left and right hemispheres [49]-[51]. This could explain why we obtained fewer misregistrations in lateral and frontal WM structures when the hemispheres were separately registered (approach (iii)) relative to whole-brain-FA registration (approach (ii)) (Fig. 6a, column 5). However, the separate registration of individual hemispheres can also increase misregistrations on midsagittal sections (Fig. 6a, column: 7). Notably, misregistrations resulting from approach (iv) resembled those of approach (iii) (Fig. 6a, column 11). By combining individual hemisphere registrations with complementary contrasts (i.e. approach (iv)), misregistrations were minimized (Fig. 6b).

### Influence of Spatial Registration on FA-VBS Results using TBSS as a Reference

Hemispheric asymmetry of right handed healthy subjects has been analysed previously by two independent groups using FA-VBS [13], [14]. Both studies observed significant leftward FA asymmetries along the course of fiber bundles associated with the arcuate fascicle (Table 4). However differences in their results were most likely due to different normalisation approaches. In our study, we demonstrated clear left-greater-than-right FA differences, such as the C-shaped leftward FA asymmetry along the course of the central segment of the arcuate fascicle, detected by both FA-VBS and TBSS (Fig. 4). However, our results also support the hypothesis (see, e.g., [15]) that systematic misregistration between groups could lead to apparent FA-group-differences when FA-VBS is used (Fig. 8). The correspondence between FA-VBS and TBSS statistical maps depended strongly on the registration approach employed for FA-VBS (Fig. 5). We identified apparent FA differences in the FA-VBS results that were (a) specific to the applied registration approach, (b) located within or next to regions susceptible to misregistration, and (c) not detected by TBSS (Fig. 8). Moreover, we found that more voxels were susceptible to misregistration when the mismatch between TBSS and FA-VBS was bigger (Fig. 9). The best correspondence between TBSS and FA-VBS results was found when the separate-hemisphere multi-contrast approach (iv) was used. FA-VBS using approach (iv) also revealed additional left-greater-than-right differences adjacent to the sensorimotor cortex (Fig. 4), which is in accordance with cerebral asymmetries associated with handedness [13].

### Additional Methodological Considerations

To identify regions prone to misregistration, we compared the single-contrast registration approaches (i-iii) to the multi-contrast, separate-hemisphere approach (iv) using the *F*-value method. This approach was motivated by the fact that approach (iv) showed the smallest amount of misregistration (Fig. 6 and 7). In this context, we would like to point out that this method is not sensitive to regions prone to misregistrations which are common to both the single-contrast (i-iii) and multi-contrast registration approach (iv). Residual registration errors in the multi-contrast approach could be further reduced by combining approach (i) and (iii) in an iterative manner [28], [40], or by combining multiple contrasts during the minimisation process [25], [26].

Methods to correct for multiple comparisons differ between TBSS and FA-VBS analysis. TBSS uses a permutation method for inference on statistical maps [52], whereas SPM uses parametric statistics [53]. We investigated the influence of these different inference statistics on the reported findings (data not shown) and found that they were negligible compared to the effects of interest, i.e. effect due to different registration approaches. Therefore, in this study we used the default statistics of each method, i.e. parametric statistics for SPM and non-parameteric statistics for TBSS. For the TBSS analysis the “threshold-free cluster enhancement” method is recommended [43], whereas in FA-VBS p-values may be uncorrected or corrected using, for example, the false discovery rate or family-wise error rate correction [54], [55]. Here we used uncorrected p-values to be more sensitive to false FA asymmetries resulting from systematic misregistrations. Since correction of multiple comparisons is generally recommended for VBS analyses [56], we performed the analysis shown in Figure 5 using two different p-values (p<0.01 and p<0.001) and found that the result did not depend on the choice of p-value.

FA-VBS results can also depend on the choice of the smoothing kernel [57]. In this study, we chose a fixed smoothing kernel of 4mm, which corresponds to the “typical intrinsic smoothness of the final skeletonized FA data” in TBSS [15] and therefore renders our FA-VBS and TBSS results comparable. Using other smoothing kernels in the FA-VBS analyses could compromise the comparability with TBSS. Accordingly, the dependence of the registration approaches on the smoothing kernel was not discussed here and should be investigated in a separate study.

Note that in Figure 8 we used prior knowledge in conjunction with F-maps that revealed misregistrations to find candidates for apparent FA asymmetries. In particular, we assumed that apparent FA differences due to systematic misregistrations are expected to be at the edge of WM matter structures, where misregistrations will lead to the highest FA differences (see Fig. 1).

A complete assessment and discussion of hemispheric asymmetries is beyond the scope of this study, which primarily focuses on methodological issues regarding spatial registration methods. Finally, the registration approaches discussed in this paper were based on the spatial normalisation routine in SPM [38]. However, other registration approaches are available that might outperform the suggested registration approach. For example, see Klein et al. [45] for a comparison of established registration approaches or Zöllei et al. [48], Ashburner et al. [58] for newly suggested approaches.

### Impact on Future Studies

The investigation of cerebral asymmetries may help to better understand human brain function [16], [29], [30], [59]-[65]. For example FA-VBS may detect correlations between WM microstructure and functional asymmetries such as handedness [13], [66]. Extrapolating from the present results on hemispheric asymmetries, we hypothesize that for whole brain FA-VBS analyses the registration method will affect the detected FA differences and multi-contrast registration will also be advantageous. Furthermore, different registration approaches or TBSS may help to disentangle FA differences due to misregistrations from microstructural differences. Note that in a recent whole-brain FA-VBS study we showed that white matter degenerations associated with juvenile myoclonic epilepsy [28] could be better detected using multi-contrast, iterative registration than single-contrast registration. Further studies will be necessary to refine the best registration approach for whole-brain FA-VBS analyses.

### Conclusion

In conclusion we report that FA-VBS results depend strongly on the employed registration approach. Specifically, we showed that the multi-contrast single-hemisphere registration is superior to single-contrast registration approaches resulting in the least systematic misregistrations and the best correspondence with TBSS results. Furthermore, given an optimal registration method, FA-VBS might be sensitive to legitimate anatomical FA asymmetries in regions beyond the central WM pathways assessed by TBSS.
